# Association among character attributes and mental health among the staff in Medical Sciences Kermanshah University in 2015

**Published:** 2015

**Authors:** A Ziapour, N Kianipour

**Affiliations:** * Social Development & Health Promotion Research Center, Kermanshah University of Medical Sciences, Kermanshah, Iran,; ** Research Committee, Kermanshah University of Medical Sciences, Kermanshah, Iran

**Keywords:** mental health, personality traits, employees

## Abstract

Workplace stress affects the employee’s mental health and customers can run their occupational safety and health care centers in damage. The employees who are sent to the work place have different characters and different events happen in their living event. The present research proposed to study the relation among character attributes and mental health among the staff in Medical Sciences Kermanshah University, the study being performed for the year 2015. In a cross-correlation, 270 employees working in Medical Sciences Kermanshah University in 2015 were randomly selected and neo personality traits and mental health question sheet were augmented with Goldenberg. Data in SPSS 21 are explained with utilizing detailed statistics, correlation coefficient of Pearson and Regression testing. Based on the analysis of solidarity, of conscientiousness (R =0/ 332, p <0/ 001), OCD (R =0/ 221, p <0/ 001) and extraversion (p <0/ 001, R= 115/ 0, the employee’s mental health showed a meaningful positive relationship (p <0/ 001). The outcomes of the regression study explained that, among the 5 parameters of character attributes, conscientiousness and psychoneurosis have two variables 14.08 percentage change criterion variables (mental health) to remarkably prophesy staff (p <0/ 001).

It seemed to support an increased employment rate and provide psychiatric and psychological counseling for employees with improved facilities and, their income could improve their general health and thus improve the capability of the state of health services provided.

## Introduction

**Mental health**

In fact, the aspect of the concept of health is according to techniques and strategies of preventing the development of mental sickness; moreover, therapy and recovery are used [**1**]. also, the psychic strength of the successful performance of mental function results in fruitful activities, satisfying relations by the others, the capacity to adapt to change and cope with adversity [**2**,**3**]. The mental health refers to the feeling of wellbeing and ensures the effectiveness, self-reliance, and self-actualization capacity, competition, intellectual and emotional potential, etc. [**4**,**5**]. Today, in most countries, the attempt to industrialization and increasing immigration and urbanization could be seen in these fundamental differences of the day to day tension and problems of mental health and, increased social communities and the world observed significant differences in stem epidemiology of the disease. The health needs such as mental illness and early dying are not the early reason of inability now [**6**]. according to the results of mental confusions via one of the very significant and most meaningful components of the overall burden , illness are predicted that in 2020 the portion of the overall burden of psychic confusions and neurological diseases will increase by 50% to 15% of the duration , 10.5 percent of the cases being alone straw convention [**7**,**8**]. Therefore, the way to psychic well-being in every area of private, familiar and employment matters [**9**,**10**]. Considering that mental health is one of the pillars of health, life requires a helpful, efficient and satisfying person by promoting psychic well-being in particular population segments that require an efficient and effective dynamics, improvement and development of the community [**11**,**12**]. Being one of the states of mental health, it is essential to consider the attributes of the character. The character is the most fundamental concept of psychology. Psychology is a field that examines the attributes of individual characters, both emotional and behavior, and are usually fixed, predictions being followed in daily life [**13**]. With regard to the very essential component of paced organization, human subjects with their different personalities, motivations, abilities, attitudes, beliefs and ideas are really important components that make up the human personality, expectations, etc., and expect people and that organization to be determined [**14**]. Thus, without a detailed information of the people, a fit between personality and job creation won't be reasonable and it will be a difficulty that many organizations grapple with [**15**,**16**]. Since the work force is one of the biggest sources of funding for each organization, health having the capacity of enhancing the efficiency, any planning and investment in the sector leads to the maintenance and improving of the employees’ health and it can ultimately direct to a raised efficiency and recover the investment associated with it. An effective management can't be performed without the consideration to the employee's health, if that enterprise forgets the human dimension of that job environment and leads it to an acceptable level of performance by adopting different methods, the staff organizations have features such as abnormalities, physical and nervous diseases expecting as the chance to exist to their psychological distress negligence, absence, rumors and character assassination [**17**,**18**]. Therefore, the mental health staff uses appropriate methods to eliminate disruptive factors of mental health and efficient deployment of forces into companies was affected by character attributes [**19**-**22**]. Mental health staff and managers monitor such practices, quality of life, efficiency administration, and in special the characteristics of the affected person [**22**-**24**]. Personality traits, including (OCD, extraversion, agreeableness, openness and conscientiousness), in turn, play a role in mental health, followed by the plays [**19**,**25**,**26**]. Research has shown that employees, have, beside their external characteristics, a far better relationship with the other employees, resulting in an improved performance and a better health system [**19**-**22**,**25**,**27**]. On the other hand, according to the categories of individuals, an organization can help organizations achieve efficiency [**28**,**29**]. Achieving the organization objectives represents an action based on force capabilities in that fulfillment from the tasks assigned and the versatility of a changing environment. In the meantime, based on the manager, the people's characterization make the staff work such as to provide in these matters that they like and thereby their efficiency & productiveness of the structure adds to this [**30**]. This study is in line with the existing research gap, and, based on the Medical Sciences Kermanshah University, there has been no study on the relation among employees. Moreover, 5 parameters of personality and mental health in Medical Sciences Kermanshah University have been analyzed to evaluate the relation among the five-factor model personality (neurosis, extraversion, receptivity, agreeability and painstakingly), all dealing with mental health.

## Research Methodology

**Research methods, population, and sample:** This is an expressive cross-correlation study. The study sample was represented by all the employees working in different administrative groups (contract, treaty, and formal), and an upper secondary school with 872 people was formed in the Medical Sciences Kermanshah University headquarters and Health Services, in 2015. According the sampling table by Krejcie & Morgan, the volume of this unit is of 270 (130 males and 137 females). A simplistic casual polling was performed by the utilizing of a table of random numbers. The inclusion criteria are according to a consent to take part in the research, have at least a secondary school degree and a work experience of two years in the University and want to participate in the study.

**Measuring tool**

**Questionnaire:** To define the people's attributes, a questionnaire containing demographic characteristics (sex, age, education and work experience), was used.

**High 5 Character parameters Questionnaire**: to measure the high 5 Character, the **(NEO-PI-R) NEO Personality Inventory-Revised** was used.

A personality test is according to a parameter study performed in 1985 by Paul T. Costa and Robert Armak McCrae from the Health National Institute in the Aging Center of Research at Baltimore, Maryland [**31**]. This test of 60 items is according to Likert scale (1 = forcefully oppose, 2 = oppose, 3 = slightly, 4 = accept, 5 = forcefully accept) and was designed as one of the Big Five personality 12 Items (OCD, extraversion, agreeability, receptivity and painstakingly), measured and calculated scores for each factor and the five scores obtained [**32**-**34**]. A narrative content to Costa and McCrae (1992), was reviewed having a 90/ 0 reliability for neuroticism, extraversion equal to 0/ 78 to 0/ 76 to openness, agreeableness equal to 0/ 86 and 0/ 90 responsibility to report [**31**]. This test was performed after being translated and adapted from Persian [**35**]. Gross Carpet in Iran (2001) confirmed the Five parameters of question sheet structure as a whole and its internal consistency with coefficients reported of Cronbach for the main factors were of 0/ 86, 0/ 73, 0/ 56, 0/68 & 0/87 [**36**]. The reliability test utilizing alpha coefficient of Cronbach for the United States is utilized in an example of 0/ 93 OCD, extraversion 0/ 87, graceful of 0/ 89, 0/ 76 and flexibility or conscientiousness and the task of 0/ 86, respectively [**37**]. Kyamehr validated this questionnaire on 380 students of Tehran University, the questionnaire with Cronbach’s alpha coefficient of internal consistency being between 0/ 54 to 0/ 79 acquired [**38**]. Hejazi et al. (2002) described a value of alpha 74% [**39**]. In the reserach, alpha coefficient of Cronbach values for OCD were the following: 0/ 91, extraversion 0/ 78, agreeableness 0/ 76, experience of 0/ 73 ( receptivity to experiment) and understanding 0/ 86 respectively. The options mentioned by Mathias questionnaire before were between 0 and 4 (strongly disagree = 0, disagree = 1, somewhat = 2, somewhat agree = 3, strongly agree = 4). The general range of Mathias questionnaire was between 0 and 240.

**General Health Questionnaire Goldenberg** (28 - Health Questionnaire = GHQ): General Health Questionnaire by Goldberg was established in 1972 and was built on a screening questionnaire, being according to self-report clinical methods set out to trace those who have the disorder [**40**-**43**] The question sheet of General Health contained 28 items in the form of an option. The seven-item questionnaire contained the four Somatic Symptoms subscales, Stress and Restlessness, Political Dysfunction and Depression Measures [**40**] Scoring with a 4-point Likert scale (0 = none to 3 = to submit a more than usual) took place. Each person in this test received four numbers and the numbers are achieved an overall score. In 2004, Noorbala noted the psychometric characteristics of inner texture GHQ, having = 0/ 83 [**44**]. In 1999, the physical flexibility of the system, GHQ 85/ 0 was highlighted by utilizing alpha coefficient of Cronbach for somatic symptoms, anxiety and insomnia, 0/ 87, 0/ 79 social dysfunction, symptoms of serious depression of 0/ 91 and the whole numbers of 0/83 representing the public health [**45**]. In 2004, Rumi carried out the test on 116 Iranian students and the questions analysis in the questionnaire, Cronbach’s alpha reaching a value of 91/ 0. In 1994, Jacob validated the test on 625 residents in city and village regions and Some’esara used the simple Likert scoring method of sensitivity and specificity of the test in the best cut off point of 23, respectively of 0/5/86 and 0/ 82 [**46**] The Mathias’ questionnaire range was between 0 and 84. Regarding Cronbach’s alpha, the reliability was of 0/ 898 in the ccurrent research, informing that the questionnaire had a good reliability and internal solidarity. To gather information, the necessary permits are received from the Science and Technology Department of the University of Origin & from the responsible staff from the university. At first, this study purpose was to describe people and ethics by filling in a questionnaire and ensuring the necessary part concerning the private data confidentiality. Moreover, a consent to associate in the sample of the study was signed. The consent of the characters to engage in the research and they should at least a high school diploma qualification, a work experience of two years in the University were respectively delivered in an record. After complement the question sheet, the data were analyzed and descriptive statistics (frequency, mean) and probable statistic was used. The mental health of multiple linear regressions is utilized to define the relation among the 5 dimension of personality variables. An important grade of interest in the research is few than 0/ 05. The consideration of information analysis is done by using SPSS21 software.

**Findings**

In the research of 223 patients, 137 (51.3%) are men & 130 (48.71%) were females The mean life of the unit is of 0/6/7 ± 38. 41 to 46 years group, should the greatest rate of 64, 24%. Regarding learning, the samples with the bachelor’s degree were of 64% (171 persons). Greatest of the units (89, 33.3%) were between 11 & 14 years of service. The mean sample and regular variation was of 8.5 years ± 17 obliteration of the type of employment relationship, more samples (108, 40. 4%) being contracted (**Table 1**).

The outcomes explained that the character attributes are associated with the greatest and lowest factors, “character attribute neurosis” by an average ± SD of 0.29 ±3.49 and “personality traits of extraversion” with a mean ± SD of 0.37 ± 3.34 (**Table 2**).

In connection with the mental health samples, the outcomes explained that the greatest rate of “social dysfunction”. The average and regular variation was of 0.40 ± 3.31 and the lowest grade for “depression” with the average and regular variation was of 0.39 ± 2.60 (**Table 2**).

Regarding the relation among the character attributes, the mental health of employees and significant were of (R = 0.328, p < 0.001). Also, the conscientiousness personality trait most related to OCD was R = 0.332, p <0. 001 and the character traits of the lowest possible relationship with OCD experience (R = 0.072, p>0. 001) (**Table 3**).

**Table 1:** Characteristics of individual samples

Frequency (percent)	Groups	Demographic
137 (3/ 51)	Man	Gender
130 (7/ 48)	Woman	
66 (7/ 24)	≥ 30	Age (years)
41 (4/ 15)	35-31	
42 (7/ 15)	40-36	
64 (24)	45-41	
47 (6/ 17)	50-46	
7 (6.2)	≤ 50	
18 (7/ 6)	Diploma	Education
35 (1/ 13)	Degree	
171 (64)	License	
28 (5/ 10)	Master’s degree or higher	
15 (6.5)	PhD	
10 (7.3)	≥ 5	Work experience (years)
19 (1/ 7)	10-6	
89 (33.3)	15-11	
70 (2/ 26)	20-16	
79 (6/ 29)	≤ 21	

**Table 2:** Index of statistical variables

Variable	Index scale	The mean (SD)	Minimum, maximum
Personality traits	OCD (N)	49/ 3 (29/ 0)	42/ 2.4
	Openness O	49/ 3 (27/ 0)	50/ 2.4
	Agreeableness A	44/ 3 (31/ 0)	58/ 2.4
	Loyalty C	38/ 3 (31/ 0)	25/ 2.4
	Extroversion E	34/ 3 (37/ 0)	25/ 2.4
	General characteristics	43/ 3 (16/ 0)	92/ 2.87/ 3
Mental Health	Impairment of social functioning	31/ 3 (40/ 0)	67/ 1.4
	Physical symptoms	03/ 3 (41/ 0)	86/ 1.4
	Anxiety and sleep disorders	99/ 2 (56/ 0)	67/ 1.4
	Depression	60/ 2 (39/ 0)	43/ 1.57/ 3
	Mental health	98/ 2 (28/ 0)	04/ 2.57/ 3

As it can view in **Tab. 2**, the findings showed that the greatest numbers of the five personality traits, employees, the personality trait neuroticism, with a average deviation criterion (mean = 3.49, SD = 0.29) and the lowest index of the personality traits of extroversion with a mean deviation criterion being of (mean = 3. 34, SD = 0.37). The mean total measure personality traits employees was of (mean = 3.43, SD = 0.16), meaning the personality traits the personnel desired. Also, the mental health variable, the highest mean and SD criteria related to social dysfunction (Mean = 3.31, SD = 0.40) and the lowest mean and SD criteria for depression (Mean = 2.60, SD = 0.39), was allocated. In the mean deviation criterion the mental health staff range was (Mean = 2.98, SD = 0.28).

**Table 3:** Correlation coefficients of Pearson among personality and students happiness

data	Free changeable	related changeable	Coefficient of Correlation	SIG (2-tailed)
1	Conscientiousness (conscientiousness)	Mental Health	0.332**	0.000
2	Neuroticism (OCD)	Mental Health	0.221**	0.000
3	Extroversion (extroversion)	Mental Health	0.115**	0.041
4	Openness to experience (the experience of)	Mental Health	0.099**	0.105
5	Agreeableness (Agreeableness)	Mental Health	0.072**	0.242
6	(Total) Personality characteristics	(Total) Mental Health	0.328**	0.000
Correlation was significant at 0.01 (2-tailed)				

As presented in **Tab. 3** (R = 0, p <0.001). The personality trait conscientiousness presented the strongest relationships with the mental health (R = 0. 332, p <0.001). In addition, the personality trait agreeableness had the lowest possible correlation with the mental health (R = 0. 072, p>0.001). Generally, the relation among the character attributes and for example, the mental health of employees, is significant.

**Table 4:** Explanation of the employee's mental health based on personality traits

Statistical indicators Model 1	Multiple correlation coefficient	The determination coefficient R2	The determination coefficient net R	SEM models
Amounts	0/ 385	0/ 148	0/ 142	0/ 26121

**Table 5:** Explanation of the coefficients of the variables affecting the mental health of employees

Sig	t	Standardized Coefficients	Unstandardized Coefficients		Variables
		Beta	Std. Error	B	
0.000	5.315	-	0.254	1.384	(Constant)
0.000	5.552	0.316	0.050	0.280	Conscientiousness Duty
0.001	3.423	0.195	0.057	0.197	Neuroticism OCD

 The multiple regression analysis method (Stepwise) according to **Table 4**, and in anticipation of the total mental health variables remained significant in this next step. The multiple correlation coefficient was equal to R = 0/ 385, determination coefficient R2= 0/ 148 and the determination coefficient of the net R2 = 0/ 142 was obtained. The important impact of the changeable in the model, up to about 14.8% of the variation can be described by the mental health staff. The standard Beta variables coefficients to be considered were the following: conscientiousness with Beta β =0/ 316 and neuroticism with beta β=0/ 195. The highest correlation in predicting the mental health scores were, in fact, owing to the low score and indicated a high level of psychic well-being and mental health of individuals. In fact, painstakingly and neurosis was positively correlated by the mental health level.

## Discussion

The purpose of the research is to study the relation among the personality and the employee’s mental health of in Medical Sciences Kermanshah University. The outcomes explained where is a necessary relation among neurosis and mental health (R = 0/ 221). The findings of most early analysis in that field [**47**-**52**] were highlighted. Taking into account these findings, it could be shown that people with neuroticism subscale, the high scores people are anxious, depressed, have a sense of guilt in a variety of fields, low self-esteem, are woven, unreasonable, shy and moody [**53**]. They were too inclined to irrational beliefs, and are not able to control their impulsivity and stress. According to this explanation, it can be assumed that employees with high neuroticism, stress and distress as of the veins are also expected to reduce the overall health. The outcomes of this research explained that there is a significant positive relation among responsibility and extroversion features and components of the mental health staff. It could be stated that the relation to responsibility and the employee’s mental health is significant (R = 0/ 332). The results of the researches [**48**, **50**, **53**, and **54**] were consistent. It could be assumed that person by a great conscientiousness, are careful, meticulous, punctual, reliable and able to manage the impulses favorably [**18**,**50**]. So, it could be arranged that the optimal ability to control impulses, stresses and coping with social situations in people with higher scores in the subscales of receptivity to knowledge and responsibility earned more areas to improve their overall health provision. The outcomes explained that the relationship among extroversion and a substantial positive relationship by the mental health staff was (R = 0/ 115). This signifies that person that should a great mental health extraversion score better. Also, by increasing the extroversion personality characteristics, the rates health mental increase as well. It can be concluded that extraverts are happy, energetic and optimistic people, willing to communicate by others, for them life is movement and emotion of desire and a demand to the environments and positive emotions [**55**]. According to this explanation, it can be deduced that the combination of persons has social skills and Communicate with other psychic well-being and more. The outcomes of this section of the analysis study were underlined by A. et al., 2009; Goodwin and Friedman, 2006; Ansell et al., 2007 [**19**,**49**,**55**]. According to Gupta & Kumar, the extroversion leads to enjoyment and support in group exercises. Therefore, it could be considered that the extroverted people are happier because they share their inner feelings with others and their minds are occupied with different things and they cannot just focus on the negative experiences [**56**]. Larsen & Keterlaar also suggested that extroverted people more than introverted people respond positively to stimuli and are stronger. Therefore, when these people find themselves in enjoyable situations they express more positive feelings [**57**]. The outcomes of the regression analysis in the research explained that the components of the five personality factors that were predicted for the component of overall health of employees are the following: conscientiousness and OCD. In fact, based on the prioritization of the value of predictive factors, conscientiousness and neuroticism are the very significant parts in predicting mental health. The openness and agreeableness variables were importantly correlated by mental health in the research. The result of some researches [**58**-**59**] were consistent at this respect.

## Conclusion

The highest and lowest average characteristics of workers are “neuroticism” and “extroversion”, respectively. The rate of mental health, as well as “social dysfunction” more and “depression” are the least met among the employees. The personality trait of painstakingly and neurosis were two dimensions significantly associated with mental health and mental health among predictor variables. This seems to support an increased employment and provide psychiatric and psychological counseling for employees with improved facilities and their income can improve their general health and thereby improve the quality of health services provided by them.

The going research faced several constraints. In the research, the use of self-report data was collected, but the method might have affected the correctness of the outcomes. In addition, by owing to the subjects of the research, the Medical Sciences Kermanshah University employees, the results cannot be generalized to other employees of other universities of medical sciences. 
